# Heterologous expression of HCoV-HKU1 ORF 7b by mouse hepatitis virus protects against severe disease during murine infection

**DOI:** 10.1371/journal.ppat.1013148

**Published:** 2025-12-05

**Authors:** Chaminda D. Gunawardene, Isha Pandey, Shruti Chatterjee, Yoatzin Peñaflor-Téllez, Abby Odle, Abbey N. Warren, Adriana Messyasz, Ricardo Rajsbaum, Alan Sariol, Lok-Yin Roy Wong

**Affiliations:** 1 Center for Virus-Host Innate Immunity, Rutgers New Jersey Medical School, Newark, New Jersey, United States of America; 2 Department of Microbiology, Biochemistry and Molecular Genetics, Rutgers New Jersey Medical School, Newark, New Jersey, United States of America; 3 Department of Medicine, Rutgers New Jersey Medical School, Newark, New Jersey, United States of America; 4 Molecular and Genomics Informatics Core Facility, Rutgers New Jersey Medical School, Newark, New Jersey, United States of America; 5 Department of Medicine, Washington University School of Medicine, St. Louis, Missouri, United States of America; KU: The University of Kansas, UNITED STATES OF AMERICA

## Abstract

Coronaviruses express a repertoire of accessory proteins for evading host immune responses. A small internal (I) accessory gene overlaps with the nucleocapsid (N) gene in an alternative reading frame of viruses that belong to the genus *Betacoronavirus*. Previous studies reported that I proteins of SARS-CoV (9b), MERS-CoV (8b) and SARS-CoV-2 (9b) inhibit type I interferon (IFN-I) expression through distinct mechanisms and have different roles in pathogenesis. In contrast, the functions of the I proteins of human coronaviruses HCoV-HKU1 (7b) and HCoV-OC43 (8b) have not been previously reported. Although HCoV-HKU1 and HCoV-OC43 predominantly cause common cold in healthy adults (common cold CoVs, CCCoVs), susceptible individuals infected with these viruses can develop severe disease. The lack of robust reverse genetic systems, tissue culture and animal models limit the study of HCoV-HKU1 and HCoV-OC43 pathogenesis. Here, we examined how the heterologous expression of the HCoV-HKU1 and HCoV-OC43 I proteins impact pathogenesis in a mouse model of infection using a prototypic betacoronavirus. We inserted the I gene of HCoV-HKU1 (ORF 7b) and HCoV-OC43 (ORF 8b) independently into the genome of a neurotropic strain of mouse hepatitis virus (J2.2). J2.2 infection is well characterized with clearly defined immune responses which allows the study of these genes in the context of authentic coronavirus infection. We showed that ORF 7b of HCoV-HKU1, but not ORF 8b of HCoV-OC43, ameliorated MHV-J2.2 pathogenesis while ORF 8b of MERS-CoV exacerbated disease. The presence of HCoV-HKU1 ORF 7b decreased virus titers and cytokine expression while ORF 8b of MERS-CoV led to increased immune cell infiltration and virus titers in mice after J2.2 infection. Moreover, proteins expressed by ORF 7b of HCoV-HKU1 and ORF 8b of HCoV-OC43 showed different patterns of subcellular localization. Overall, our findings suggest that the I genes of different betacoronaviruses play unique roles in pathogenesis.

## Author summary

Factors governing the differences in the pathogenicity between highly pathogenic human coronaviruses (SARS-CoV, MERS-CoV and SARS-CoV-2) and seasonal coronaviruses (HCoV-HKU1 and HCoV-OC43) are not completely understood. The difference in pathogenicity is at least in part contributed by the accessory genes encoded by these two groups of human coronaviruses. Heterologous expression of accessory genes in a model betacoronavirus provides an isogenic background for the direct comparison of viral proteins encoded by various coronaviruses. In this study, we compared the virulence of MHV-J2.2 that express I genes encoded by other betacoronaviruse. We demonstrated that ORF 7b of the seasonal coronaviruses HCoV-HKU1 but not the ORF 8b of the highly pathogenic MERS-CoV contributes to attenuated disease in the context of MHV-J2.2 infection, highlighting that virus-specific functions of accessory genes encoded by different HCoVs.

## Introduction

Nine coronaviruses have been reported to infect humans up to date [1–9]. Among these 9 human coronaviruses (HCoVs), 5 belong to the genus *Betacoronavirus*, namely HCoV-OC43, HCoV-HKU1, severe acute respiratory syndrome coronavirus (SARS-CoV), Middle East respiratory syndrome coronavirus (MERS-CoV) and SARS-CoV-2. SARS-CoV, MERS-CoV and SARS-CoV-2 are known to cause acute respiratory distress syndrome (ARDS) in humans and are known as highly pathogenic HCoVs [1,10,11]. HCoV-HKU1 and HCoV-OC43 infection in humans primarily result in mild self-limiting upper respiratory tract infection with common cold symptoms that resolve without major complications [12,13]. Although HCoV-HKU1 and HCoV-OC43 are considered common cold CoVs (CCCoVs), susceptible populations may also develop pneumonia upon lower respiratory tract infection [3,14]. The mechanism of pathogenesis after infection with HCoV-HKU1 and HCoV-OC43 has not been fully understood.

Coronaviruses express different accessory proteins that are known to modulate host immune responses and contribute to pathogenesis [15–19]. Although accessory proteins are conserved among members of the same subgenus, this may not be true for the genus. The three highly pathogenic HCoVs belong to two different subgenera *Sarbecovirus* (SARS-CoV and SARS-CoV-2) and *Merbecovirus* (MERS-CoV) while CCCoVs HCoV-HKU1 and HCoV-OC43 are members of the genus *Embecovirus* which also includes mouse hepatitis virus (MHV) that infects rodents. These viruses express a diverse repertoire of accessory proteins that may contribute to disease severity upon infection. In addition, highly pathogenic HCoVs often encode extra accessory proteins compared to CCCoVs [20]. This prompted the possibility that the difference in pathogenicity between highly pathogenic HCoVs and CCCoVs may be attributed in part to the accessory proteins encoded by distinct HCoVs.

While betacoronaviruses encode accessory proteins that differ among subgenera, a small “internal” (I) accessory gene that overlaps with the nucleocapsid (N) gene in an alternative reading frame at the 3’ end of the genome is encoded by all betacoronaviruses [21]. Expression of the I proteins is dependent on the subgenomic RNA (sgRNA) of N as no transcriptional regulatory sequence (TRS) specific to the I genes has been identified. Synthesis of the I proteins is initiated by leaky ribosomal scanning of the N sgRNA at an alternative (+1) reading frame. Previous studies showed that the I proteins of SARS-CoV (protein 9b), MERS-CoV (protein 8b) and SARS-CoV-2 (protein 9b) antagonize interferon (IFN) induction through distinct mechanisms [18,22,23]. Furthermore, we previously showed that protein 8b of MERS-CoV and protein 9b of SARS-CoV-2 play opposite role in modulating pathogenesis in experimentally infected animals [19]. However, the functions of the I proteins of other betacoronaviruses including HCoV-HKU1 (protein 7b) and HCoV-OC43 (protein 8b) have not been previously described. Also, the evolutionary relationships of the I proteins encoded by the different betacoronaviruses are not fully resolved. In addition, SARS-CoV-2 was shown to encode two unrelated I proteins, 9b and 9c using alternative reading frames that overlap with the N gene. These observations show that different I proteins may exist depending on coronavirus lineage and the exact location of its gene relative to the N gene. Thus, the designation of the “I” gene or protein should be interpreted with caution as multiple proteins can be generated from alternative reading frames within the N locus.

Since robust reverse genetic systems, tissue culture and animal models are not currently available for HCoV-HKU1, we dissected the functions of its I protein in the context of murine infection by a prototypic mouse hepatitis virus (MHV). MHV strain J2.2 is neurotropic and causes demyelinating encephalomyelitis. J2.2 infection has been well characterized with clearly defined immune responses [24–28]. Previous reports demonstrated the feasibility of this model for studying accessory proteins of highly pathogenic HCoVs including SARS-CoV and MERS-CoV [19,29,30]. In addition, the J2.2 model serves as a platform for comparing the role of I proteins of betacoronaviruses in an isogenic virus background due to their virus-specific roles reported previously [19,31]. In this study, we investigate the function of the I proteins of HCoV-HKU1 and HCoV-OC43 in relation to pathogenesis. We introduced the HCoV-HKU1 ORF 7b and HCoV-OC43 ORF 8b into J2.2. In addition, we included J2.2 encoding MERS-CoV ORF 8b generated previously for comparison [19]. Introduction of these ORFs into J2.2 resulted in protein expression in infected cells. Consistent with previous reports, introduction of MERS-CoV ORF 8b to J2.2 resulted in enhanced disease in infected mice [19,30]. However, the presence of HCoV-HKU1 ORF 7b attenuated J2.2 infection with improved survival in infected mice, while the HCoV-OC43 ORF 8b did not contribute to significant changes in disease outcomes after J2.2 infection. Further analysis revealed that the presence of MERS-CoV ORF 8b promotes neutrophil infiltration and virus replication in the brain while the HCoV-HKU1 ORF 7b decreased virus replication in the brain. Infection of bone marrow-derived macrophages (BMDM) with MHV-J2.2 encoding the HCoV-HKU1 ORF 7b results in decreased cytokine expression. Consistently, transcriptomic analysis indicates that ORF 7b of HCoV-HKU1 reduces gene expression related to inflammatory response, IFN signaling, antiviral response and macrophage activation. Moreover, the I proteins of HCoV-HKU1 and HCoV-OC43 exhibit different patterns of localization in infected cells, suggesting a possible mechanism for the distinct disease outcomes. Taken together, our results revealed the contrasting roles of betacoronavirus I proteins in contribution to pathogenesis using a heterologous CoV infection model.

## Results

### Generation and validation of J2.2 viruses expressing I proteins of HCoV-HKU1 and HCoV-OC43

**Fig 1:**
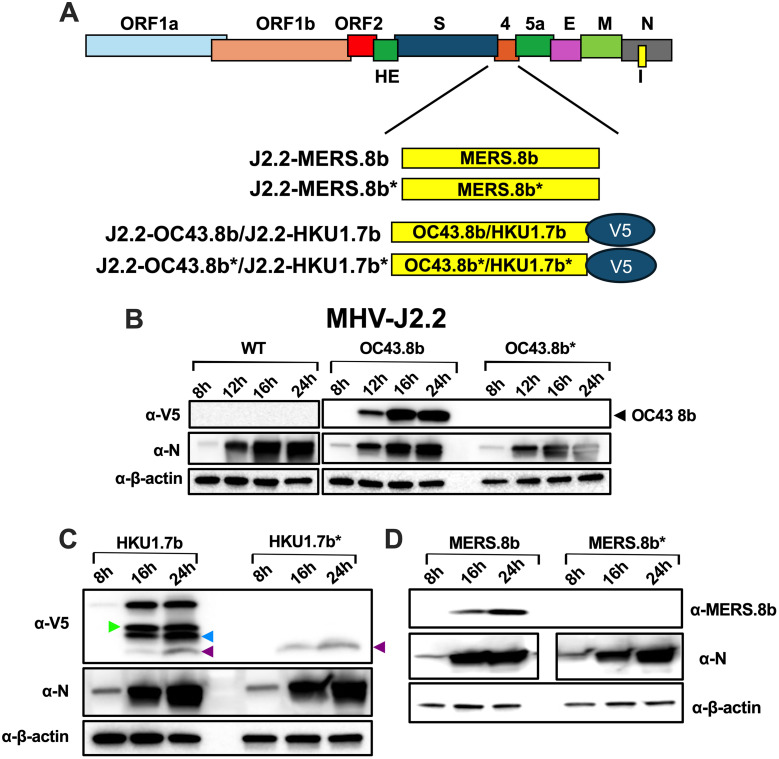
Verification of I protein expression in J2.2-infected cells. **(A)** Schematic diagram illustrating the introduction of I genes and the corresponding control sequences to replace ORF 4 of J2.2. **(B-D)** 17Cl-1 cells were infected with the indicated viruses at a multiplicity of infection (MOI) of 0.01. Infected cells were harvested at the indicated time point. Cell lysates were subject to SDS-PAGE. The protein 8b of HCoV-OC43 (B) and protein 7b HCoV-HKU1 (C) were detected with an anti-V5 antibody (α-V5). **(D)** MERS-CoV protein 8b was detected with an anti-8b antibody (α-8b) previously generated [18]. Viral nucleocapsid protein (α-N) and β-actin (α-β-actin) were probed to control for virus replication and protein amount, respectively. The putative start codons for the three protein products (green, blue and purple arrowheads) are shown in **S1 Fig**. Images are representative of three independent experiments.

To characterize the role of HCoV-HKU1 and HCoV-OC43 I proteins, we replaced open reading frame (ORF) 4 of J2.2 with the I genes of HCoV-HKU1 and HCoV-OC43 independently to generate J2.2 expressing the ORF 7b of HCoV-HKU1 (J2.2-HKU1.7b) and ORF 8b of HCoV-OC43 (J2.2-OC43.8b), respectively. The absence of ORF 4 is not known to affect virus replication or result in attenuation of virulence [32]. A V5 epitope tag was included at the C-terminal end of the I protein sequences for detection. To control for the addition of the I gene sequences, we further replaced ORF 4 with sequences identical to that of the I genes of HCoV-HKU1 and HCoV-OC43 except with nonsense mutations that abolish the expression of the I proteins to generate J2.2-HKU1.7b* and J2.2-OC43.8b* respectively. Furthermore, we included J2.2 expressing MERS-CoV ORF 8b (J2.2-MERS.8b) and the corresponding control virus (J2.2-MERS.8b*) generated as previously described [19] (**Fig 1A**). The detailed sequences and the positions of nonsense mutations introduced to the control viruses are shown in **S1 Fig**.

We next assessed I protein expression in infected cells (**Fig 1B-1D**). Cells infected with all J2.2 viruses exhibit increasing nucleocapsid (N) protein expression (α-N) from 8 hours post infection (hpi) to 24 hpi, indicating active virus replication. Wild-type (WT) J2.2-infected cells did not express any V5-tagged proteins (α-V5) (**Fig 1B, left panel**). J2.2-OC43.8b-infected cells expressed the V5-tagged 8b protein as expected with increasing expression from 8-16 hpi and remained relatively constant from 16-24 hpi. Cells infected with J2.2-OC43.8b* did not express any V5-tagged proteins (**Fig 1B, right panel**). Interestingly, cells infected with J2.2-HKU1.7b expressed multiple V5-tagged proteins with increasing expression from 8-16 hpi and remain at peak expression till 24 hpi, suggesting that multiple proteins are expressed by the inserted HCoV-HKU1 ORF 7b at start codons downstream of the first methionine residue in the context of J2.2 infection, which were detected by the C-terminal V5 tag. The top V5 band corresponds to the molecular weight of the full-length protein 7b of HCoV-HKU1. The putative start codons for the smaller bands are illustrated in **S1 Fig**. V5-tagged proteins were not detected in J2.2-HKU1.7b*-infected cells except for the one with the smallest molecular weight and lowest expression level (**Fig 1C, purple arrowhead**). The band intensity of this protein is much lower than other protein products, suggesting that a minor protein of small molecular weight is expressed by the HCoV-HKU1 ORF 7b during infection of J2.2-HKU1.7b and J2.2-HKU1.7b*. Due to its low level of expression, we reason that this minor protein product would not complicate our analysis in a biologically relevant manner and that the phenotypic differences observed between J2.2-HKU1.7b and J2.2-HKU1.7b* can be attributed to other major protein products (HCoV-HKU1 7b proteins) but not this minor protein. Consistent with previous reports [19], MERS-CoV protein 8b expression (α-8b) was detected in cells infected with J2.2-MERS.8b but not that infected with J2.2-MERS.8b* using an antibody targeting protein 8b that was raised previously [18] (**Fig 1D**).

### HCoV-HKU1 ORF 7b attenuates MHV infection *in vivo*

**Fig 2:**
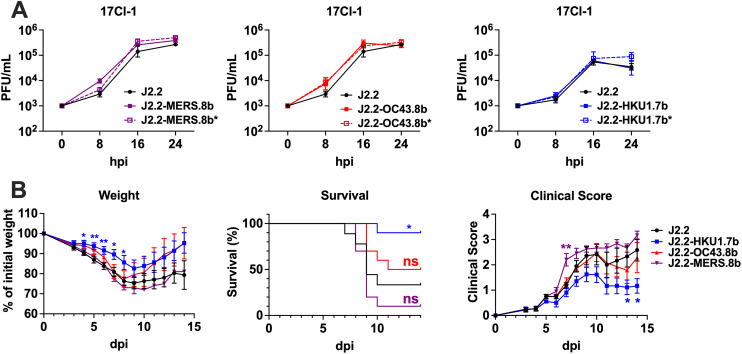
Effects of I protein on virus replication and disease outcomes. **(A)** 17Cl-1 cells were infected with WT J2.2 (J2.2, black solid line), J2.2 expressing the protein 8b of MERS-CoV (J2.2-MERS.8b, purple solid line) or J2.2 control virus for the protein 8b of MERS-CoV (J2.2- MERS.8b*, purple dashed line) (left panel); WT J2.2 (J2.2, black solid line), J2.2 expressing the protein 8b of HCoV-OC43 (J2.2-OC43.8b, red solid line) or J2.2 control virus for the protein 8b of HCoV-OC43 (J2.2-OC43.8b*, red dashed line) (middle panel); WT J2.2 (J2.2, black solid line), J2.2 expressing the protein 7b of HCoV-HKU1 (J2.2-HKU1.7b, blue solid line) or J2.2 control virus for the protein 7b of HCoV-HKU1 (J2.2-HKU1.7b*, blue dashed line) (right panel) at an MOI of 0.01. Cells and supernatant were collected at the indicated time points for plaque assay to determine virus titers (n = 3 for each data point). Data are representative of three independent experiments. Data points are shown as geometric mean ± geometric SD. **(B)** Percent of initial weight (left panel), survival (middle panel) and clinical scores (right panel) of C57BL/6 mice intracranially infected with 750 PFU of WT J2.2 (black), J2.2-HKU1.7b (blue), J2.2-OC43.8b (red) or J2.2-MERS.8b (purple). Data are pooled from at least two independent experiments (n ≥ 9 for each group). Data points are shown as mean ± SEM for the weight curve and the panel for clinical score. *P < 0.05, **P < 0.01 by Student’s t test at each time point as compared to J2.2. The color of asterisk represents the group of interest in the weight curve and the panel for clinical score. The P value in the survival curve was determined with logrank (Mantel-Cox) test followed by Bonferroni’s correction for multiple comparisons. The survival curve of J2.2 is significantly different from J2.2-HKU1.7b but not J2.2-OC43.8b and J2.2-MERS.8b. Font color represents statistical significance of the correspondingly colored group compared to J2.2. *P < 0.05.

**Fig 3:**
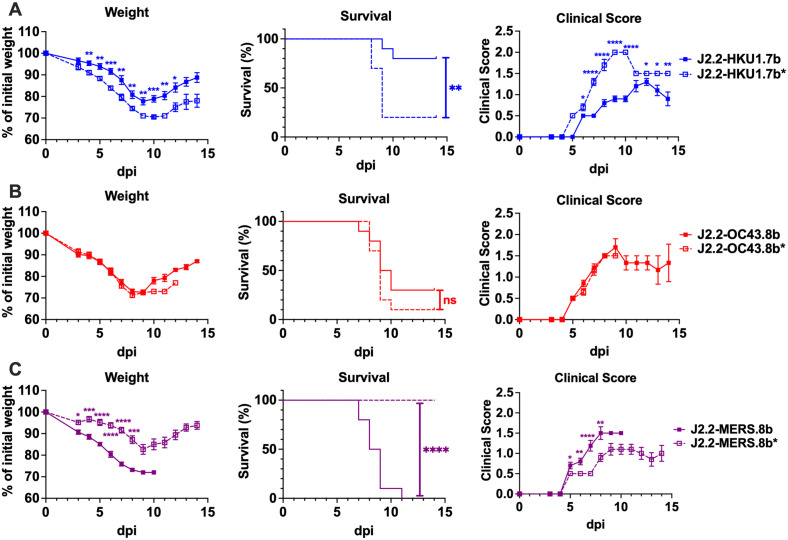
The I proteins of HCoV-HKU1, HCoV-OC43 and MERS-CoV play unique roles in J2.2 pathogenesis. Percent of initial weight (left panel), survival (middle panel) and clinical scores (right panel) of C57BL/6 mice intracranially infected with 750 PFU of **(A)** J2.2-HKU1.7b or J2.2-HKU1.7b*; **(B)** J2.2-OC43.8b or J2.2-OC43.8b*; **(C)** J2.2-MERS.8b or J2.2-MERS.8b*. Data are pooled from at least two independent experiments (n = 10 for each group). Data points are shown as mean ± SEM for the weight curve and the panel for clinical score. *P < 0.05, **P < 0.01, ***P < 0.001, ****P < 0.0001 by Student’s t test at each time point. The P value in the survival curve was determined with log-rank (Mantel-Cox) test. **P < 0.01, ****P < 0.0001.

We next investigated if the I genes play any role in regulating virus replication in a mouse fibroblast cell line 17Cl-1, which is reported to induce minimal interferon expression after MHV infection [33]. In general, J2.2 expressing the different I genes did not contribute to significantly altered replication as compared to WT J2.2 and the corresponding control viruses except at 8 hpi, where J2.2-MERS.8b demonstrated a slight growth advantage (**Fig 2A**). To evaluate the function of the I genes in relation to pathogenesis, we infected C57BL/6 mice with WT J2.2, J2.2-HKU1.7b, J2.2-OC43.8b and J2.2-MERS.8b. In agreement with previous reports, mice infected with J2.2-MERS.8b developed more severe disease as compared to WT J2.2 [19,30]. However, J2.2-HKU1.7b caused attenuated disease with reduced weight loss and clinical score and increased survival in mice as compared to those infected with WT J2.2 (**Fig 2B**). To further confirm the impact of the I genes on disease outcomes after J2.2 infection, we infected mice with J2.2 expressing the different I genes and the corresponding control viruses. We again observed that mice infected with J2.2-HKU1.7b developed attenuated disease as compared to those infected with J2.2-HKU1.7b* (**Fig 3A**) while mice infected with J2.2-OC43.8b and J2.2-OC43.8b* developed similar clinical disease (**Fig 3B**). However, J2.2-MERS.8b caused significantly more severe disease with increased weight loss, mortality and clinical score as compared to those infected with J2.2-MERS.8b* (**Fig 3C**). These data indicate that ORF 7b of HCoV-HKU1 attenuates J2.2 infection while ORF 8b of MERS-CoV exacerbates disease outcomes after infection, consistent with previous reports [19,30]. In addition, we observed that while most of the animals infected with J2.2-HKU1.7b* and J2.2-OC43.8b* succumbed to infection, all those infected with J2.2-MERS.8b* survived. Therefore, we compared the virulence of the three control viruses (J2.2-HKU1.7b*, J2.2-OC43.8b* and J2.2-MERS.8b*) to WT J2.2. J2.2-HKU1.7b*, J2.2-OC43.8b* and WT J2.2 caused similar disease in infected mice while J2.2-MERS.8b* is significantly attenuated as compared to WT J2.2 and the control viruses (**S1 Fig**). These data underscore the importance of using viruses that control for the sequences being introduced for comparison, as introduction of sequences may have various effects on virulence.

To further investigate the attenuating role of HCoV-HKU1 ORF 7b, we introduced HCoV-HKU1 ORF 7b sequence and the corresponding control sequence into a neurovirulent strain of MHV (JHM), the parental virus of J2.2 [34], to generate JHM-HKU1.7b and JHM-HKU1.7b*, respectively (**S2A Fig**). In addition, we generated a virus that expresses the JHM I gene (ORF 7b) in place of ORF 4 (JHM-JHM.7b) to control for the effects of genomic locus on I gene function. Expression of the corresponding V5-tagged proteins for these viruses is shown in **S2B Fig**. Consistent with the findings in J2.2, JHM-HKU1.7b significantly attenuated JHM infection as compared to WT JHM and JHM-HKU1.7b*. Furthermore, JHM-JHM.7b-infected mice also developed severe disease (**S2C Fig**). These data confirmed the specific attenuating role of HCoV-HKU1 ORF 7b as attenuation was not observed with the introduction of ORF 7b of JHM at the ORF 4 locus.

Our previous study showed that deletion of JHM ORF 7b attenuated infection as JHM protein 7b contributes to virion packaging and assembly [31]. Therefore, it is unclear if the attenuating role observed with HCoV-HKU1 ORF 7b was mediated through the impairment of JHM protein 7b function by HCoV-HKU1 7b proteins. To address this, we compared virus replication in cells infected with JHM-HKU1.7b, JHM-HKU1.7b* or the previously generated JHM with its protein 7b deleted (JHM-JHM.7b*). We reason that JHM-HKU1.7b would show reduced replication as in the case of JHM-JHM.7b* if the HCoV-HKU1 7b proteins negatively impact the function of JHM protein 7b. However, we observed that JHM-HKU1.7b replicated to significantly higher titers than JHM-JHM.7b* but not JHM-HKU1.7b* at 12 and 16 hpi (**S2D–F Fig**). These data suggest that the attenuation observed in the presence of HCoV-HKU1 7b proteins is not mediated through the impediment of the JHM protein 7b function.

### HCoV-HKU1 ORF 7b mediates modest reduction in virus replication and dampens inflammatory responses in the brain

**Fig 4:**
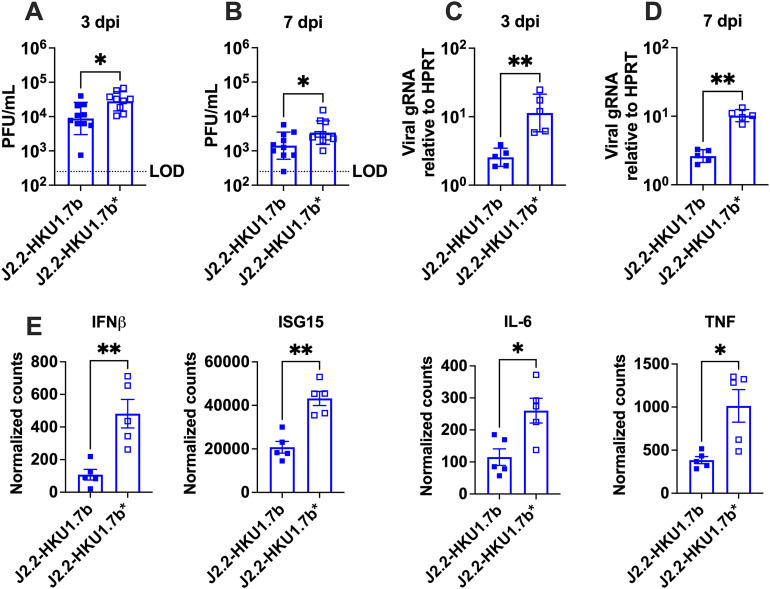
J2.2-HKU1.7b replicates less efficiently in the brain. C57BL/6 mice were infected with 750 PFU of J2.2-HKU1.7b or J2.2-HKU1.7b* intracranially. The brains of infected mice were collected and homogenized at 3 (A, C) and 7 (B, D) dpi. **(A, B)** Infectious virus titers were determined by plaque assay in HeLa-MVR cells. **(C, D)** Expression levels of viral genomic RNA were assessed by qPCR. Data are pooled from two independent experiments in A and B and are representative of two independent experiments in C and D. Data points are shown as geometric mean ± geometric SD. Each point represents data obtained from an individual mouse. *P < 0.05, **P < 0.01 by Student’s t test. **(E)** Normalized gene counts obtained from RNA-seq data (S3 Fig). Each point represents data obtained from an individual mouse. Data points are shown as mean ± SEM. *P < 0.05, **P < 0.01 by Student’s t test.

Based on these results, we determined the basis of attenuation afforded by the HCoV-HKU1 ORF 7b. We assessed virus replication in the brain of mice infected with J2.2-HKU1.7b or J2.2-HKU1.7b*. Consistent with the observed attenuation, mice infected with J2.2-HKU1.7b have a subtle but significant reduction in virus titers in the brains as compared to those infected with J2.2-HKU1.7b* at 3 and 7 days post infection (dpi) (**Fig 4A and 4B**). Similarly, we also identified significantly less viral genomic RNA (gRNA) in J2.2-HKU1.7b-infected brains (**Fig 4C and 4D**). To characterize how the HCoV-HKU1 ORF 7b attenuates J2.2 infection, we performed RNA sequencing on J2.2-HKU1.7b- and J2.2-HKU1.7b*-infected brains at 3 dpi. We observed 602 differentially expressed genes (523 upregulated and 79 downregulated; adjusted p values<0.05) in mice infected with J2.2-HKU1.7b as compared to those infected with J2.2-HKU1.7b* (**S3A Fig**). Genes related to cytokine storm, IFN signaling and neutrophil degranulation were significantly downregulated in J2.2-HKU1.7b-infected mice (**S3B-D Fig**). Furthermore, we performed Ingenuity Pathway Analysis (IPA) to reveal significantly altered biological pathways (**S3E Fig**). In addition to the pathways shown in **S3B-D Fig**, J2.2-HKU1.7b-infected mice express lower levels of genes involved in ISGylation signaling pathway, antiviral response and macrophage activation pathway. Notably, the normalized gene counts for IFNβ, ISG15, IL-6 and TNF were significantly lower in J2.2-HKU1.7b-infected mice compared to those infected with J2.2-HKU1.7b* (**Fig 4E**). These data suggest that J2.2-HKU1.7b-infected mice mount modest antiviral and inflammatory responses with attenuated disease as compared to those infected with J2.2-HKU1.7b*.

**Fig 5:**
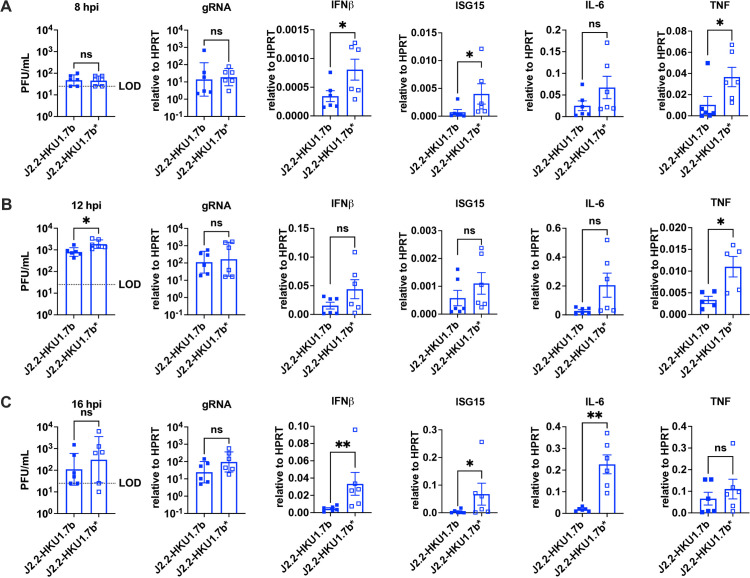
Decreased cytokine expression in J2.2-HKU1.7b-infected BMDM. BMDM isolated from C57BL/6 mice were infected with J2.2-HKU1.7b or J2.2-HKU1.7b* at an MOI of 0.01. Infected BMDM were collected for determination of virus titers and cytokine expression by qPCR at 8 **(A)**, 12 **(B)** and 16 **(C)** hpi. Data are pooled from two independent experiments. Each point represents data obtained from an individual biological replicate. Data points for virus titers and gRNA are shown as geometric mean ± geometric SD. Data points for cytokine expression are shown as mean ± SEM. *P < 0.05, **P < 0.01 by Student’s t test.

Next, we investigated if the HCoV-HKU1 ORF 7b plays a role in suppressing cytokine induction. BMDMs isolated from C57BL/6 mice were infected with J2.2-HKU1.7b or J2.2-HKU1.7b* to assess virus titers and cytokine induction (**Fig 5**). Overall, we did not observe significant differences in virus titers between J2.2-HKU1.7b- and J2.2-HKU1.7b*-infected BMDMs at 8 and 16 hpi despite a subtle increase in J2.2-HKU1.7b*-infected BMDM at 12 hpi. The levels of viral genomic RNA were also not significantly different in BMDM infected with J2.2-HKU1.7b or J2.2-HKU1.7b* (**Fig 5**). However, the expression of IFNβ and ISG15 were significantly diminished at 8 and 16 hpi in J2.2-HKU1.7b-infected BMDM (**Fig 5A and 5C**). In addition, significantly less induction of TNF was detected at 8 and 12 hpi with lower IL-6 expression at 16 hpi in J2.2-HKU1.7b-infected BMDM. Collectively, these data suggest that the HCoV-HKU1 ORF 7b attenuates J2.2 infection by inducing less cytokine expression.

### MERS-CoV ORF 8b but not HCoV-HKU1 ORF 7b promotes virus replication and immune cell infiltration in the brain

**Fig 6:**
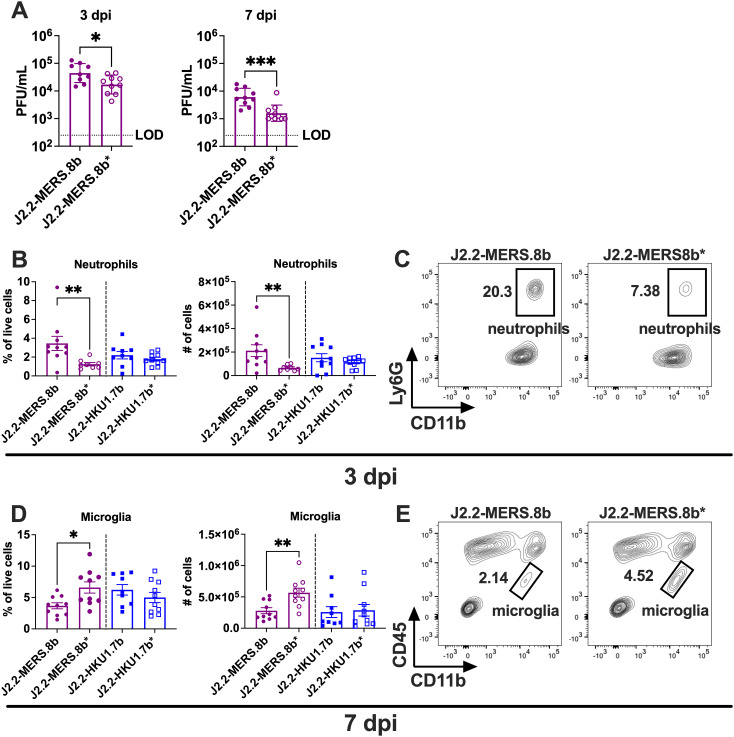
Changes in immune cell populations in J2.2-MERS.8b-infected brain. C57BL/6 mice were intracranially infected with 750 PFU of J2.2-MERS.8b or J2.2-MERS.8b* **(A)**; J2.2-MERS.8b, J2.2-MERS.8b*, J2.2-HKU1.7b or J2.2-HKU1.7b* **(B-E)**. The brains were harvested at 3 and 7 dpi for determination of infectious virus titers (A) and flow cytometric analysis of immune cell infiltration (B-E). Data in A are pooled from two independent experiments. Each point represents data obtained from an individual mouse. Data points are shown as geometric mean ± geometric SD. *P < 0.05, ***P < 0.001 by Student’s t test. **(B, D)** Frequency (left panel) and number (right panel) of neutrophils (B) and microglia (D) in infected brains are illustrated. Data are pooled from two independent experiments. Each point represents data obtained from an individual mouse. Data points are shown as mean ± SEM. *P < 0.05, **P < 0.01 by Student’s t test. **(C, E)** Representative flow plots of neutrophils (C) and microglia (E) in J2.2-MERS.8b- (left panel) or J2.2-MERS.8b*-infected (right panel) brains.

We and others demonstrated previously that MERS-CoV ORF 8b enhances disease after J2.2 infection [19,30]. Here, we further characterize and compare the role of MERS-CoV ORF 8b and the HCoV-HKU1 ORF 7b in modulating disease outcomes after J2.2 infection at the cellular level. We observed that the brains of mice infected with J2.2-MERS.8b harbors more infectious virus as compared to those infected with J2.2-MERS.8b* at 3 and 7 dpi (**Fig 6A**). This is in agreement with previous reports [19,30] and our observation (**Fig 3**) that MERS-CoV ORF 8b promotes severe disease with concomitant increase in virus replication in the brain in the context of J2.2 infection. Since J2.2 infection is known to recruit immune cells to the brain, we next evaluated the levels of different immune cell populations in the brains of mice infected with the I gene-encoding viruses and their corresponding control viruses. Mice infected with J2.2-MERS.8b showed an increase in frequency and number of neutrophils at 3 dpi (**Fig 6B and 6C**), suggesting that the presence of MERS-CoV ORF 8b promotes the recruitment of pro-inflammatory neutrophils, possibly a consequence of increased virus replication, which contributes to severe disease. We also observed lower frequency and number of microglia at 7 dpi in the brains of J2.2-MERS.8b-infected mice as compared to those infected with J2.2-MERS.8b* (**Fig 6D and 6E**). This suggests that MERS-CoV ORF 8b may reduce microgliosis which leads to worse disease outcomes as microglia are important for protection during acute MHV infection and recovery after virus clearance [24,25]. In contrast, we did not identify significant changes in the frequency and number of neutrophils or microglia or other immune cells in the brains of mice infected with J2.2-HKU1.7b compared to those infected with J2.2-HKU1.7b* (**Figs 6 and** **S4**). These data provide a plausible explanation for the role of MERS-CoV ORF 8b but not the HCoV-HKU1 ORF 7b in exacerbating J2.2 infection.

### I proteins of HCoV-HKU1 and HCoV-OC43 exhibit different patterns of cellular localization

**Fig 7:**
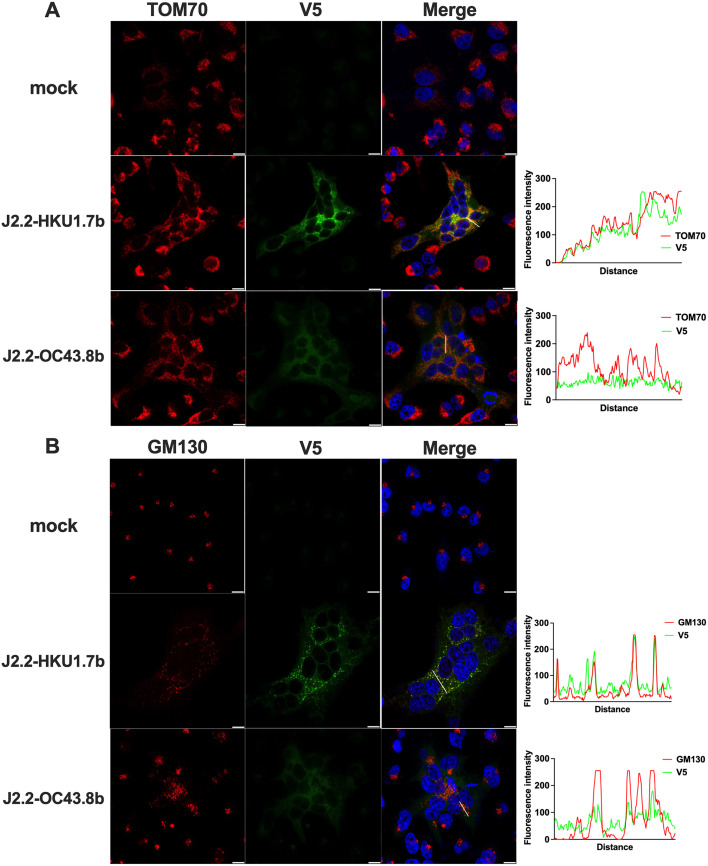
Distinct cellular localization of the I proteins of HCoV-HKU1 and HCoV-OC43. HeLa-MVR cells were infected with J2.2-HKU1.7b or J2.2-OC43.8b at an MOI of 0.01. Cells were washed and fixed at 16 hpi. The I protein signal was determined with an anti-V5 antibody (V5, green) while TOM70 (A) or GM130 (B) were stained in red. Intensity of fluorescence signals for the I protein (V5) and TOM70 (A) or GM130 (B) in infected cells along the indicated white line in the merged images are shown. Representative images from two independent experiments are shown. Scale bar = 10 µm.

From our results, the HCoV-HKU1 ORF 7b but not the HCoV-OC43 ORF 8b ameliorates J2.2 infection (**Figs 3 and 4**). To determine a mechanism for this difference in infection outcomes, we first assessed subcellular localization of the I proteins expressed by HCoV-HKU1 ORF 7b and HCoV-OC43 ORF 8b. To test this, HeLa cells expressing murine carcinoembryonic antigen-related cell adhesion molecule (CEACAM1) (HeLa-MVR), the receptor for MHV were infected with J2.2-HKU1.7b or J2.2-OC43.8b. Infected HeLa-MVR were processed for immunofluorescence with an antibody against V5 tag to probe for the I proteins. HCoV-HKU1 7b proteins formed regions of strong fluorescence signals and punctate structures while diffused fluorescence signal across infected cells was observed for the protein 8b of HCoV-OC43 (**Fig 7**). Since MERS-CoV protein 8b and SARS-CoV-2 protein 9b were shown to interact with mitochondrial protein translocase of outer mitochondrial membrane 70 (TOM70) to varying extent [19,22,35], we stained for TOM70 in J2.2-HKU1.7b- and J2.2-OC43.8b-infected cells. As shown in **Fig 7A**, overlapping V5 and TOM70 fluorescence signals were observed in J2.2-HKU1.7b-infected cells but not in cells infected with J2.2-OC43.8b, suggesting that HCoV-HKU1 7b proteins co-localize with TOM70. We further stained for Golgi matrix protein GM130 to determine if the I proteins of HCoV-HKU1 and HCoV-OC43 localize at the Golgi complex. We observed moderate overlapping between the V5 and GM130 signals in J2.2-HKU1.7b-infected cells. However, co-localization between the protein 8b of HCoV-OC43 and GM130 was not detected (**Fig 7B**). Interestingly, condensed and perinuclear GM130 signal were observed in mock-infected cells while diffused GM130 signal was detected across J2.2-HKU1.7b-infected cells with the loss of perinuclear localization, suggesting that HCoV-HKU1 7b proteins may disrupt the Golgi complex. Similarly, we identified diffused GM130 signal in some J2.2-OC43.8b-infected cells albeit not as prominent as those infected with J2.2-HKU1.7b. Overall, these data suggest that the I proteins of HCoV-HKU1 and HCoV-OC43 demonstrate distinct patterns of subcellular localization and possibly interact with different host proteins, potentially contributing to the unique infection outcomes in J2.2-HKU1.7b- and J2.2-OC43.8b-infected mice (**Fig 3**).

### Comparison of predicted I protein structures for betacoronaviruses

To further investigate how the I proteins of different betacoronaviruses mediate disparate infection outcomes, we compared the sequences and structures of these I proteins (**S5 Fig**). Sequence analysis of the I protein of SARS-CoV-2, MERS-CoV, HCoV-HKU1 (full-length protein 7b), HCoV-OC43 and MHV-JHM revealed that these I proteins did not share substantial sequence similarity. However, the I proteins of HCoV-HKU1, HCoV-OC43 and MHV-JHM share a higher sequence similarity among themselves than with MERS-CoV protein 8b and SARS-CoV-2 protein 9b. Since the experimental structures of the I proteins of HCoV-OC43 and HCoV-HKU1 are not available, we used AlphaFold to predict the structure of the protein 8b of HCoV-OC43 and the full-length protein 7b of HCoV-HKU1 [36]. The predicted structure of HCoV-OC43 protein 8b consists of disordered N- and C-termini with a central domain composed of an alpha-helix (**S5B Fig**). Similarly, the C-terminus of the HCoV-HKU1 protein 7b also consists of an alpha-helix followed by a C-terminal disordered tail. In contrast, the N-terminus of HCoV-HKU1 protein 7b is composed of several alpha-helical structures connected by linker regions which are not present in HCoV-OC43 protein 8b (**S5B Fig**). We also observed extensive overlap between the C-terminus of HCoV-HKU1 protein 7b and the HCoV-OC43 protein 8b, indicating high degree of sequence and structure homology between the HCoV-OC43 protein 8b and the C-terminus of HCoV-HKU1 protein 7b (**S1B** **and** **S5B Fig**). Moreover, the predicted structures of the I proteins of MERS-CoV and JHM (protein 8b and 7b, respectively) obtained from previous studies follow a similar architecture with a central alpha-helix with disordered N- and C-terminal domains (**S5C Fig**) [19,31]. SARS-CoV-2 protein 9b also adopts a predominantly alpha-helical structure when interacting with TOM70 [22,35,37]. These data suggest that the I proteins of betacoronaviruses may share a similar core structure despite low degree of sequence similarity among subgenera but yet contribute to disparate infection outcomes.

## Discussion

Accessory proteins are the least conserved viral proteins among coronaviruses as compared to the structural and non-structural proteins, especially for viruses of different subgenera. However, betacoronaviruses of several subgenera encode an I protein as one of their accessory proteins, suggesting that the presence of an I gene and the expression of an I protein is a conserved feature among betacoronaviruses. Although the I proteins of several betacoronaviruses were reported to act as an IFN antagonist, many CoVs encode multiple IFN antagonists with redundant functions [18,38–43]. Therefore, it is unlikely that the need for IFN antagonism alone selected for the expression of the I proteins. Furthermore, the I gene is harbored within the N gene, which poses additional evolutionary constraints to the I protein, suggesting that undocumented I protein functions are important for CoV evolution. Interestingly, our previous study reported that the predicted structure of the protein 8b of MERS-CoV resembles the experimentally determined structure of the protein 9b of SARS-CoV-2 when interacting with its host protein TOM70 despite limited sequence homology [19]. This highlights the possibility that the I proteins of betacoronaviruses may form analogous structures and perform similar functions in the host during infection. Similarly, the protein 7b of HCoV-HKU1 and protein 8b of HCoV-OC43 share low degree of sequence identity with the protein 8b of MERS-CoV and protein 9b of SARS-CoV-2. Whether the I proteins of HCoV-HKU1 and HCoV-OC43 fold into structures comparable to that of the I proteins of MERS-CoV and SARS-CoV-2 requires further investigation. Although the protein 7b of HCoV-HKU1 showed signs of co-localization with TOM70 and the Golgi complex, these findings require further validation with authentic HCoV-HKU1 and pulldown assay. The interaction between the protein 7b of HCoV-HKU1 and TOM70 or Golgi complex, if validated, should be further characterized to determine the mode of binding (direct vs. indirect, mediated by other host factors) and the molecular interactions responsible for the binding. In addition, mitochondria are molecular hubs for anti-viral and cell death signaling. CoV infection is known to perturb mitochondria-mediated immune response and induce cell death [22,44]. The impact of protein 7b of HCoV-HKU1 on host signaling cascades via mitochondria, through its interaction with TOM70, if validated, may provide insights on the mechanisms of cell death and immune regulation mediated by this protein during CoV infection. Future research focusing on the structural perspectives of these I proteins will elucidate critical virus-host interactions that are important for I protein-mediated pathogenesis.

While an I protein is expressed by betacoronaviruses, their evolutionary origin remains elusive. Sequence analysis showed that these I proteins share extensive similarity within subgenus, consistent with the classification as accessory or lineage-specific protein. However, these I proteins share low levels of similarity across subgenera (**S5A Fig**). When the genomic location of these I genes were mapped relative to the N protein domain, we once again observed similar origin of these I genes relative to the N protein domains within each subgenus but not across subgenera (**S6 Fig**). Interestingly, the HCoV-HKU1 ORF 7b initiates from a region of the N gene encoding the N-terminal intrinsically disordered domain (N-IDR) of the N protein as opposed to the N-terminal domain (NTD) for ORF 7b and ORF 8b of MHV-JHM and HCoV-OC43. However, ORF 7b of HCoV-HKU1 encodes downstream start codons within regions of the N gene that encode the NTD (**S6 Fig**). This suggests that these I proteins are orthologous within subgenus but not across subgenera of *Betacoronavirus* and possibly emerged as a consequence of convergent evolution of the different subgenera.

Based on our findings, the ORF 7b of HCoV-HKU1 causes reduction in virus replication *in vivo* with concomitant decrease in cytokine expression (**Fig 4**). However, cytokine expression was decreased in J2.2-HKU1.7b-infected BMDM without significant reduction in viral RNA levels, suggesting that HCoV-HKU1 ORF 7b suppresses immune activation in these cells (**Fig 5**). Unlike J2.2-HKU1.7b, the ORF 8b of MERS-CoV enhances J2.2 infection as it suppresses IFN and cytokine expression which leads to concomitant increase in virus replication in infected mouse brains [30]. While most of the characterized I proteins act as innate immune antagonists, the protein 7b of MHV-JHM has been shown to play a role in maintaining virion integrity and assembly in addition to immune antagonism [31]. This highlights the possible pleiotropic nature of HCoV-HKU1 7b proteins in mediating virus replication, assembly and innate immune evasion in different cells. Although we showed that the presence of HCoV-HKU1 7b proteins did not significantly impair the JHM protein 7b function in virus replication in BMDM (**S1 Fig**), protein 7b of JHM also acts as an innate immune antagonist [31]. In this study, MHV protein 7b was not genetically removed from the viruses used. Thus, the presence of MHV protein 7b may complicate the *in vivo* findings. Future experiments should investigate I protein functions in the absence of MHV protein 7b expression. Moreover, a recent study showed that some cytokines mediate efficient virus replication during MHV infection [45]. Inhibiting the expression of these proviral cytokines in J2.2-HKU1.7b-infected mice, if indicated, may also lead to the concomitant decrease in virus replication and cytokine expression observed *in vivo*.

HCoV-HKU1 and HCoV-OC43 belong to the subgenus *Embecovirus*. These two viruses share highly homologous I protein sequences. Sequence alignment reveals that the protein 8b of HCoV-OC43 aligns to the C-terminus of the HCoV-HKU1 protein 7b (**S1 Fig**). Structural prediction also showed that the C-terminus of protein 7b of HCoV-HKU1 overlaps with protein 8b of HCoV-OC43 (**S5B Fig**), suggesting that the difference in the phenotype between J2.2-HKU1.7b- and J2.2-OC43.8b-infected mice maybe attributed to the N-terminal domains of the HCoV-HKU1 protein 7b, which is not present for protein 8b of HCoV-OC43. As shown in **S5 Fig**, the N-terminus of HCoV-HKU1 protein 7b loops around the C-terminal alpha helix mimicking that of HCoV-OC43 protein 8b. We reason that the N-terminus of the HCoV-HKU1 protein 7b may alter its binding to host target as compared to HCoV-OC43 protein 8b lacking the extended N-terminus, therefore attenuating J2.2 and JHM infection. In addition, a previous study demonstrated that the N-terminus of the protein 8b of MERS-CoV is critical for IFN antagonism and enhanced disease after J2.2 infection [30], hinting a role of the N-terminal domain in mediating I protein function. Future experiments truncating the N-terminus of HCoV-HKU1 protein 7b and extending the N-terminus of HCoV-OC43 protein 8b are warranted to determine to role of the extended N-terminus in mediating protection against severe disease. Furthermore, this study also reported the presence of multiple epitope-tagged protein products expressed by the ORF 8b of MERS-CoV in the context of J2.2 infection [30]. We did not detect multiple protein bands in J2.2-MERS.8b-infected cells in this study due to the use of an antibody that targets the N-terminus of MERS-CoV protein 8b [18]. Similarly, we detected multiple protein products in J2.2-HKU1.7b-infected cells when an antibody targeting the C-terminal epitope tag was used (**Fig 1C**). The faster-migrating bands likely represent protein products in which translation initiates at alternative start codons downstream of the first start codon (**S1 Fig**). In particular, a minor protein product of small molecular weight was present in J2.2-HKU1.7b*-infected cells (**Fig 1C**). Based on these observations, we postulate that the phenotypic differences observed between J2.2-HKU1.7b and J2.2-HKU.7b* are likely contributed by the other proteins synthesized with HCoV-HKU1 ORF 7b. Future experiments are warranted to investigate the functions of these protein isoforms expressed by HCoV-HKU1 ORF 7b.

MHV-J2.2 is a neurotropic strain of MHV that causes infection of the central nervous system (CNS). This study investigates the role of I proteins of HCoV-HKU1 and HCoV-OC43 in pathogenesis, both of which are respiratory HCoVs. The mismatch in the site of infection (CNS vs airway) and tissue-specific immune responses may not reflect the phenotype of the I proteins in natural (airway) infections. Furthermore, protein 8b of MERS-CoV is reported to play opposite roles in pathogenesis in the context of MERS-CoV and J2.2 infection. We previously showed that the presence of protein 8b prevents lethal disease after MERS-CoV infection but contributes to enhanced disease in the context of J2.2 infection [19]. In fact, the role of protein 8b in exacerbating J2.2 infection has been independently reported in a separate study [30], suggesting that the I protein of HCoVs may contribute to pathogenesis in a virus-specific context. Combining with this study, future investigation in the context of authentic HCoV-HKU1 infection would offer critical insights in the virus- and/or tissue-specific role of its I proteins. The use of authentic HCoV-HKU1 remains a technical challenge as it is reported to only replicate in primary human airway cells [46], rendering it challenging for propagation and genetic manipulation.

In conclusion, we compared the I proteins of three HCoVs in relation to pathogenesis in the context of J2.2 infection. We characterized the impacts of the different I proteins on the virological properties of MHV-J2.2 and the host immunological changes in experimentally infected animals. These results further affirm the unique roles of the I proteins of various CoVs in pathogenesis and reinforce the notion that accessory proteins are important in modulating CoV life cycle in a virus-specific fashion.

## Materials and methods

### Ethics statement

All animal studies were approved by the Institutional Animal Care and Use Committee (IACUC) of Rutgers University and follow guidelines of the Guide for the Care and Use of Laboratory Animals. Studies involving the use of recombinant viruses have been approved by the Institutional Biosafety Committee of Rutgers University.

### Mice, cells and viruses

Specific-pathogen-free C57BL/6 mice were purchased from Charles River Laboratories and maintained in a specific pathogen-free facility at Rutgers University under standard conditions of dark/light cycle, ambient temperature, and humidity. Five- to seven-week-old male mice were used in all experiments. Female mice exhibit similar clinical outcomes after JHMV infection, but results are less consistent. 17Cl-1 mouse fibroblasts, HeLa cells expressing the MHV receptor carcinoembryonic antigen-related cell adhesion molecule 1 (HeLa-MVR) and BHK-21 cells were grown in Dulbecco’s modified Eagle medium (DMEM) supplemented with 10% fetal bovine serum (FBS), 100 U/mL penicillin and streptomycin, L-glutamine, sodium pyruvate, HEPES, and non-essential amino acids and maintained at 37°C. BMDMs were harvested from C57BL/6 mice and differentiated in DMEM media supplemented with 10% FBS, M-CSF (20 ng/mL), 100 U/mL penicillin and streptomycin, L-glutamine, and sodium pyruvate. BMDM media were changed every other day following the fourth day of differentiation and infected at 7 or 8 days post-differentiation. A recombinant version of the neuroattenuated J2.2-V-1 variant of JHMV (MHV-J2.2) was propagated in 17Cl-1 cells. Virus titers of MHV-J2.2 were determined in HeLa-MVR cells.

### Mouse infection

For J2.2 infection, five- to seven-week-old male mice were anesthetized with ketamine-xylazine and inoculated intracranially with 750 PFU of MHV in 30 µL of DMEM. For JHM infection, five- to seven-week-old male mice were lightly anesthetized with isoflurane and inoculated intranasally with 500 PFU of MHV in 11 µL of DMEM. Mice were monitored and weighed daily following inoculation. Clinical scoring for MHV infection was based on the following criteria: 0, asymptomatic; 1, limp tail, mild hunching; 2, wobbly gait with mild righting difficulty, hunching; 3, hind-limb paresis and extreme righting difficulty; 4, hind-limb paralysis; and 5, moribund.

### Infection of cell and virus plaque assay

Cells were washed with PBS once before infection. Viruses were diluted in DMEM for infection at the indicated MOI. Cells were incubated with virus inoculum for 1 h at the conditions described below. After infection, virus inoculum was removed and DMEM supplemented with 10% FBS was added to the well and incubated until time of harvest. For plaque assay, cells were frozen and mice were euthanized and transcardially perfused with PBS at the indicated times. Organs were harvested and homogenized, while cells were thawed prior to clarification by centrifugation and titering. Virus or tissue homogenate were serially diluted in DMEM. Twelve-well plates of HeLa-MVR were inoculated with at 37°C in 5% CO_2_ for 1 h and gently rocked every 15 min. After 1 h of incubation, virus inoculum was removed and DMEM supplemented with 10% FBS was added to the well and incubated for 16 h. After 16 h, the supernatant was removed and plates were overlaid with 0.6% agarose containing 2% FBS and 0.1% neutral red and incubated for 4 h. Plaques were counted without further treatment after 4 h of incubation. Viral titers were quantified as PFU/mL.

### Generation of mutant viruses

OC43.8b, OC43.8b*, HKU1.7b or HKU1.7b* sequences were introduced into J2.2 or JHM BAC at the ORF 4 region by a two-step linear lambda red recombination process [47–49]. The first step removed and replaced the ORF 4 sequence with GalK-Kan selection marker while the second step removed and replaced the GalK-Kan selection marker with the desired insert sequences prepared by PCR amplification followed by purification. In brief, GalK-Kan selection marker was PCR amplified from pYD-C225 [49] and gel purified. Gel-purified GalK-Kan fragments were transformed into SW102 cells carrying the J2.2 BAC by electroporation, for linear lambda red recombination. Successful recombinants were selected on Kanamycin resistance culture plates. Verified recombinants carrying GalK-Kan cassette were further introduced with the corresponding inserts by electroporation for a second round of linear lambda red recombination. Successful recombinants were selected using 2-deoxy-galactose-based culture plates, and sequence identity was verified by BAC sequencing. GalK-Kan selection markers were amplified with the following primers: forward, 5’-CTCTCCTGGAAAGACAGAAAATCTAAACAATTTATAGCATTCTCATTGCTACTTTGCTCCTCTAGAGGGCAGCAAGTAGTT**cctgttgacaattaatcatcg**-3′; reverse, 5′- TACTTCGGCAAGTGCCTAAGTGTGTATGGACGGCCAGAATTAAGATGAGGTTTAGAACTAGTAATATAATCTAGAGT**ctcagcaaaagttcgattta**-3′. Lightface capital letters represent sequence flanking the area of interest in the J2.2 BAC; sequences complementary to pYD-C225 are shown as boldface lowercase letters.

Primers amplifying HKU1.7b and HKU1.7b* were as follows: forward, 5′-CTCTCCTGGAAAGACAGAAAATCTAAACAATTTATAGCATTCTCATTGCTACTTTGCTCCTCTAGAGGGCAGCAAGTAGTTatgctggaagtagaagctcctctgg-3′; reverse, 5′- AGGTTTAGAACTAGTAATATAATCTAGAGT**TTACGTAGAATCGAGACCGAGGAGAGGGTTAGGGATAGGCTTACC**caccagaggtaggggttctattgcc -3′. Primers amplifying OC43.8b and OC43.8b* were as follows: forward, 5′- TGGAAAGACAGAAAATCTAAACAATTTATAGCATTCTCATTGCTACTTTGCTCCTCTAGAGGGCAGCAAGTAGTTatggcaaccagcgtcaactgctgcc-3′; reverse, 5′- AGGTTTAGAACTAGTAATATAATCTAGAGT**TTACGTAGAATCGAGACCGAGGAGAGGGTTAGGGATAGGCTTACC**caccagaggtaggggttctattgcc-3′. The capital letters represent sequence of the area where recombination takes place (flanking ORF 4 for MHV) while the boldface capital letters represent the V5 sequence and the lowercase letters represent sequence complementary to the corresponding I protein sequences for PCR amplification with synthesized plasmids as templates.

### Recovery of recombinant viruses and virus propagation

Two micrograms of the indicated J2.2 BACs was transfected into BHK-21 cells with Lipofectamine 3000 (Invitrogen) in a six-well plate according to the manufacturer’s protocol. Cells were monitored daily for cytopathic effects (CPE). Cultures were harvested when CPE was > 50% by freezing at −80°C. MHV-J2.2 were further passaged in 17Cl-1 cells in DMEM supplemented with 10% FBS. Virus titer was determined by plaque assay as described in the previous section.

### Protein extraction and western blot analysis

17Cl-1 cells infected with J2.2 were lysed with RIPA buffer at the indicated time points and mixed with gel-loading dye for 10 min at 95°C before electrophoresis. Cell lysates were resolved by SDS-PAGE. Specific proteins were probed with the following primary antibodies: rabbit α-ORF8b polyclonal antibody [18], mouse α-N monoclonal antibody (Cat.# MABF2751; Millipore Sigma), mouse α-V5 monoclonal antibody (Cat.# R960-25; Invitrogen) or mouse α-actin monoclonal antibody (Cat.# MA1–140; Thermo Fisher Scientific). Bands were visualized with α-mouse (Cat. # 405306; BioLegend) or α-rabbit (Cat. # 406401; BioLegend) IgG secondary antibody conjugated with horseradish peroxidase (HRP) followed by substrate incubation (Cat. # 32106; Thermo Fisher Scientific).

### RNA isolation and qRT-PCR

Cells and tissues were homogenized in TRIzol (Invitrogen) for RNA isolation as specified by the manufacturer’s protocol. Isolated RNA was subject to DNase I treatment (Invitrogen) and reverse transcribed using the SuperScript IV First-Strand Synthesis System (Invitrogen). mRNA levels were determined after normalizing with HPRT by the ΔCt method. Specific primer sets used for qPCR were previously described [50,51]. Viral genomic RNA was detected with the following primers: forward, 5’-AGGGAGTTTGACCTTGTTCAG-3’; reverse, 5’-ATAATGCACCTGTCATCCTCG-3’

### RNA sequencing and analysis

RNA libraries were prepared using the NEB Next Ultra II RNA Library Prep Kit for Illumina (Catalog #E7770) with Poly(A) selection. In brief, 100 ng of total RNA in 50 μL of nuclease-free water was denatured at 65°C for 5 minutes, rapidly cooled to 4°C, and incubated at room temperature for 5 minutes with Oligo(dT) beads to capture mRNA. The beads were washed, and the bound mRNA was eluted in tris buffer. A second round of Oligo(dT) bead purification was performed, with final elution in first-strand synthesis buffer.

cDNA synthesis was carried out in two stages: first-strand synthesis, followed by second-strand synthesis, with purification using AMPure XP beads (Beckman Coulter, Cat. # A63881). The resulting cDNA underwent end-repair, adaptor ligation, and barcoding with unique i7 and i5 index primers. A subsequent round of AMPure XP bead purification was performed to refine the libraries. Library quality was assessed using the Agilent TapeStation system (Part #G2992AA) and quantified with a Qubit fluorometer. The libraries were then pooled in equimolar ratios and sequenced on the NovaSeq X Plus platform using 1.5B, 200 cycle kit with paired-end configuration, generating an average of 50 million reads per sample.

Raw transcriptome reads were assessed for quality control (FASTQC v0.11.9) and trimmed for quality and adapter contaminants (cutadapt v 3.4). Trimmed reads were aligned to the Mus musculus genome (GRCm38) using STAR (v2.7.10a), followed by transcript abundance calculation and hit count extraction with StringTie (v2.2.1) and featureCounts (v2.0.1) respectively. Hit count normalization and differential gene expression group cross-comparisons were performed using DESeq2 (v1.44.0). Significant differentially expressed gene thresholds were set at FDR adjusted p < 0.05. Qiagen IPA (v01-23–01) Expression Analysis based on experimental log ratio and a p-adjusted cutoff of < 0.05 was used for pathway analysis. Raw RNA sequencing data were deposited to NIH Gene Expression Omnibus (Project ID: GSE291309).

### Flow cytometric analysis

Animals were anesthetized with ketamine-xylazine and perfused transcardially with 10 mL PBS. Brains were removed, minced, and digested in HBSS buffer consisting of 2% fetal calf serum, 25 mM HEPES, 1 mg/mL collagenase D (Roche), and 0.1 mg/mL DNase (Roche) at 37°C for 30 min. Single-cell suspensions were prepared by passage through a 70 µM cell strainer. Cells were enumerated with Countess 3 (Invitrogen). Cells were then washed and blocked with 1 µg α-CD16/α-CD32 (2.4G2) antibody at 4°C for 20 min and surface stained with the following antibodies at 4°C for 30 min: V450 α-CD45 (clone 30-F11; BioLegend); BV605 α-CD11b (clone M1/70; BioLegend); PE α-Ly6G (clone 1A8; BioLegend); BV785 α-Ly6C (clone HK1.4; BioLegend) and LIVE/DEAD Fixable Blue Dead Cell Stain Kit (Thermo Fisher Scientific). All flow cytometry data were acquired using a BD FACS Symphony and analyzed with FlowJo software.

### Immunofluorescence

HeLa-MVR cells seeded on cover slips were mock infected or infected with J2.2-HKU1.7b or J2.2-OC43.8b at an MOI of 0.01. At 16h post infection, cells were washed with PBS and fixed with 4% paraformaldehyde for 20 min at room temperature. Cells were washed three times with PBS and permeabilized in 0.75% of Triton X-100 for 20 min. Permeabilized cells were blocked with 1% BSA for 1 h at room temperature and stained with primary antibodies (α-V5, 1:120 dilution, Invitrogen Cat. # R960-25; α-TOM70, 1:120 dilution, Abcam Cat. # ab289977) at 4 degrees overnight. Cells were washed three times with PBS the next day and stained with secondary antibodies at room temperature for 1 h. Cells on cover slips were washed three times with PBS and mounted with Vectashield (Cat. # H-1800) on slides for imaging with Leica Stellaris 8 tau-STED laser-scanning confocal microscope.

### Structure prediction and sequence alignment

AlphaFold Colab [36], a simplified version of AlphaFold v2.3.1 204, was used to generate models of the HCoV-HKU1 protein 7b and HCoV-OC43 protein 8b. Default parameters were used. Protein sequence alignment was performed with Clustal Omega multiple sequence alignment (https://www.ebi.ac.uk/jdispatcher/msa/clustalo).

### Statistical analysis

Student’s *t*-test was used to analyze differences in mean values between groups. All results are expressed as mean ± SEM except for virus titers and viral RNA levels where data are represented as geometric mean ± geometric SD. *P*-values of <0.05 were considered statistically significant. *P < 0.05, **P < 0.01, and ***P < 0.001. Differences in mortality were analyzed using log-rank (Mantel-Cox) survival tests.

## Supporting information

S1 Fig I protein and control sequences used in this study. (A) Table showing the premature stop mutations introduced to the control viruses. (B) Sequence alignment between the I proteins of HCoV-HKU1 and HCOV-OC43. Sequences were derived from GenBank (accession number AY597011 and AY391777 for the I proteins of HCoV-HKU1 and HCoV-OC43, respectively). Methionine residues highlighted in green, blue and purple in the sequence of the HCoV-HKU1 protein 7b represent the putative start of the protein products indicated by the arrowheads of the corresponding color shown in **Fig 1C**. Putative starts are predicted based on the molecular weight of the protein products. Residues converted to stop codons in J2.2-OC43.8b* and J2.2-HKU1.7b* are highlighted in red. Shades of black and gray represent identical and similar amino acid residues between the I protein of HCoV-HKU1 and HCoV-OC43, respectively. (C) Percent of initial weight (left panel), clinical score (middle panel) and survival (right panel) of C57BL/6 mice intracranially infected with 750 PFU of J2.2, J2.2-HKU1.7b*, J2.2-OC43.8b* or J2.2-MERS.8b*. Data are pooled from two independent experiments (n = 10 for each group). Data points are shown as mean ± SEM for the weight curve and the panel for clinical score.
(TIF)

S2 Fig The 7b proteins of HCoV-HKU1 attenuate JHM infection. (A) Schematic diagram illustrating the introduction of I protein and the corresponding control sequences to replace ORF 4 of JHM. (B) HeLa-MVR cells were infected with the indicated viruses at a multiplicity of infection (MOI) of 0.01. Infected cells were harvested at 16 hpi. Cell lysates were subject to SDS-PAGE. The I proteins of HCoV-HKU1 and JHM were detected with an anti-V5 antibody (α-V5). Viral nucleocapsid protein (α-N) and β-actin (α-β-actin) were probed to control for virus replication and protein amount, respectively. (C) Percent of initial weight (left panel), survival (middle panel) and clinical scores (right panel) of C57BL/6 mice intranasally infected with 500 PFU of WT JHM (black line), JHM-HKU1.7b (blue solid line), JHM-HKU1.7b* (blue dashed line) or JHM-JHM.7b* (red line). Data are pooled from at least two independent experiments (n ≥ 5 for each group). Data points are shown as mean ± SEM for the weight curve and the panel for clinical score. The P value in the survival curve was determined with logrank (Mantel-Cox) test followed by Bonferroni’s correction for multiple comparisons. The survival curve of JHM-HKU1.7b is significantly different from JHM, JHM-HKU1.7b* and JHM-JHM.7b. Font color represents statistical significance of the correspondingly colored group compared to JHM-HKU1.7b. **P < 0.01, ***P < 0.001. (D-F) BMDM isolated from C57BL/6 mice were infected with JHM-HKU1.7b, JHM-HKU1.7b* or JHM-JHM.7b* at an MOI of 0.01. Infected BMDM were collected for determination of virus titers at 8 (D), 12 (E) and 16 (F) hpi. Data are pooled from two independent experiments. Each point represents data obtained from an individual biological replicate. Data points are shown as geometric mean ± geometric SD. Statistical significance determined by one-way ANOVA with correction for multiple comparisons. *P < 0.05, **P < 0.01.
(TIF)

S3 Fig Brains infected with J2.2-HKU1.7b show downregulation of pathways related to cytokine signaling and antiviral response. C57BL/6 mice were intracranially infected with 750 PFU of J2.2-HKU1.7b or J2.2-HKU1.7b*. The brains were harvested at 3 dpi for isolation of total RNA followed by RNA sequencing. Genes with adjusted P < 0.05 were selected for further analysis. Heat maps plotting log_10_ z-score of all significantly regulated genes (A), genes involved in pathogen induced cytokine signaling pathway (B), interferon alpha/beta signaling (C) and neutrophil degranulation (D) at 3 dpi. (E) Ingenuity Pathway Analysis (Qiagen) was used to analyze altered biological pathways (J2.2-HKU1.7b vs. J2.2-HKU1.7b*) at 3 dpi. Pathways with adjusted *P* < 0.05 are considered significant. 1: Pathogen Induced Cytokine Storm Signaling Pathway; 2: Interferon alpha/beta signaling; 3: Neutrophil degranulation; 4: ISGylation Signaling Pathway; 5: Interferon gamma signaling; 6: Role of PKR in Interferon Induction and Antiviral Response; 7: IL-27 Signaling Pathway; 8: Macrophage Classical Activation Signaling Pathway.
(TIF)

S4 Fig Immune cell profiling in the brains infected with J2.2 expressing the I proteins of MERS-CoV or HCoV-HKU1. C57BL/6 mice were intracranially infected with 750 PFU of J2.2- MERS.8b, J2.2-MERS.8b*, J2.2-HKU1.7b or J2.2-HKU1.7b*. The brains were harvested at 3 and 7 dpi for flow cytometric analysis of immune cell infiltration. Frequency (left panel) and number (right panel) of macrophages (A), microglia (B) at 3 dpi and macrophages at 7 dpi in infected brains are illustrated. Data are pooled from two independent experiments. Each point represents data obtained from an individual mouse. Data points are shown as mean ± geometric SEM.
(TIF)

S5 Fig Sequence and structural comparison of betacoronavirus I proteins. (A) Sequence alignments of the indicated betacoronavirus I protein. (B) Predicted structures of the HCoV-OC43 protein 8b (left) and the HCoV-HKU1 protein 7b (middle) using AlphaFold. Structural homology shown in the superimposed structure (right). (C) Predicted structures of MERS-CoV protein 8b [19] (left) and MHV-JHM [31] (right) obtained from previous studies.
(TIF)

S6 Fig Genomic location of internal genes relative to the N protein domain for different betacoronaviruses. (A-F) The genomic location of the internal genes of representative betacoronaviruses are illustrated relative to the N gene and the corresponding protein domains. The numbers represent the first of the two consecutive codons of the N gene that constitute the corresponding codon of the internal genes (+1 reading frame relative to N). The internal genes are encompassed within the first and last indicated codons of the N gene. The corresponding N protein domains expressed by the N sequences that overlap with internal genes are indicated. The positions of alternative start codons for each internal protein are shown in A-C. N-IDR: N-terminal intrinsically disordered region; NTD: N-terminal domain; SR: Serine/Arginine rich motif; LKR: Linker region; CTD: C-terminal domain; C-IDR: C-terminal intrinsically disordered region.
(TIF)
