# Supplementary figures and images for: Heterologous expression of HCoV-HKU1 ORF 7b by mouse hepatitis virus protects against severe disease during murine infection
Source: PLoS Pathog. 2025 Dec 5;21(12):e1013148. doi: 10.1371/journal.ppat.1013148 (PMC12697948; doi:10.1371/journal.ppat.1013148)

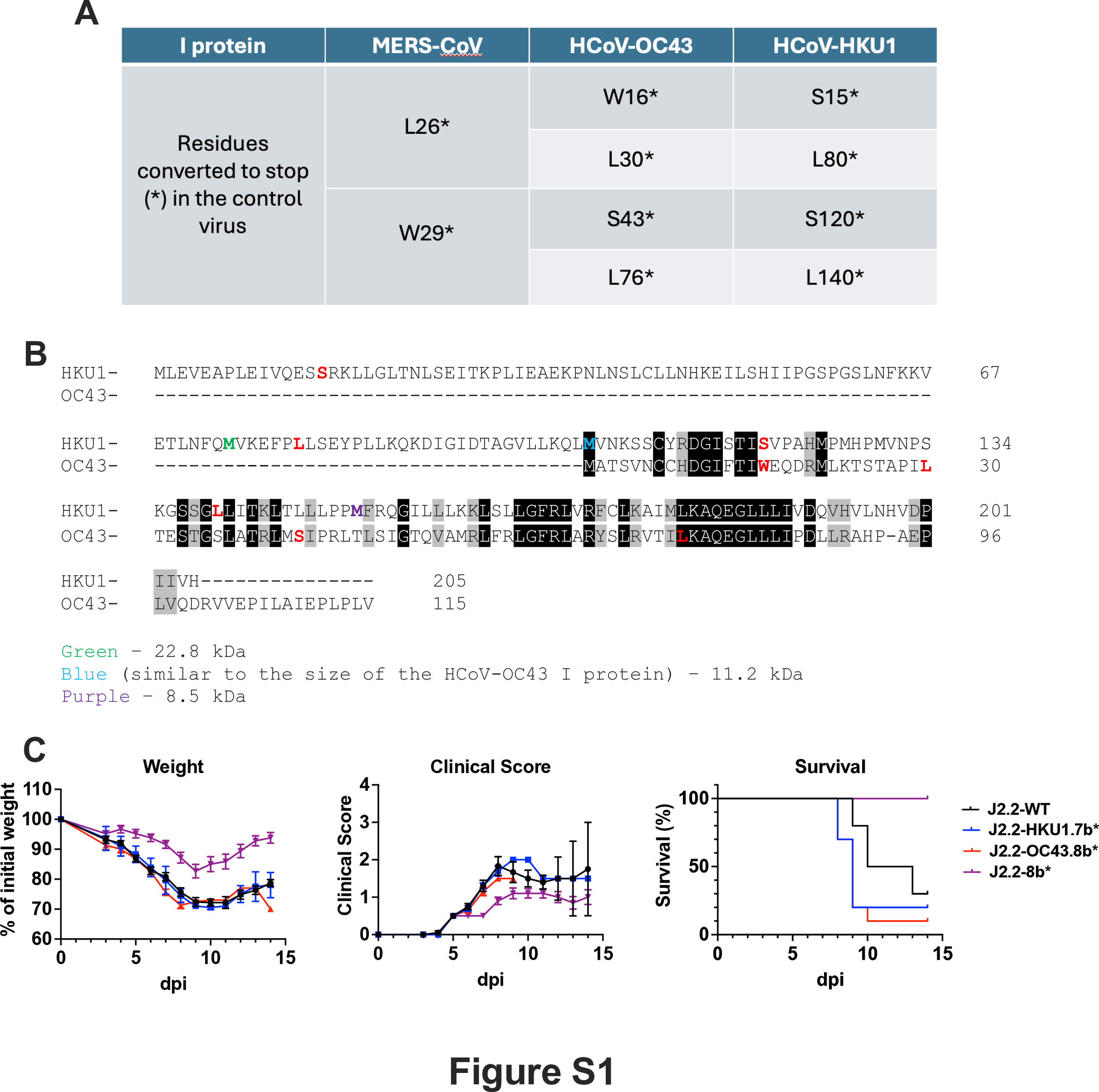

Supplement: S1 Fig — (A) Table showing the premature stop mutations introduced to the control viruses. (B) Sequence alignment between the I proteins of HCoV-HKU1 and HCOV-OC43. Sequences were derived from GenBank (accession number AY597011 and AY391777 for the I proteins of HCoV-HKU1 and HCoV-OC43, respectively). Methionine residues highlighted in green, blue and purple in the sequence of the HCoV-HKU1 protein 7b represent the putative start of the protein products indicated by the arrowheads of the corresponding color shown in Fig 1C. Putative starts are predicted based on the molecular weight of the protein products. Residues converted to stop codons in J2.2-OC43.8b* and J2.2-HKU1.7b* are highlighted in red. Shades of black and gray represent identical and similar amino acid residues between the I protein of HCoV-HKU1 and HCoV-OC43, respectively. (C) Percent of initial weight (left panel), clinical score (middle panel) and survival (right panel) of C57BL/6 mice intracranially infected with 750 PFU of J2.2, J2.2-HKU1.7b*, J2.2-OC43.8b* or J2.2-MERS.8b*. Data are pooled from two independent experiments (n = 10 for each group). Data points are shown as mean ± SEM for the weight curve and the panel for clinical score. (TIF) [file ppat.1013148.s001.tif]

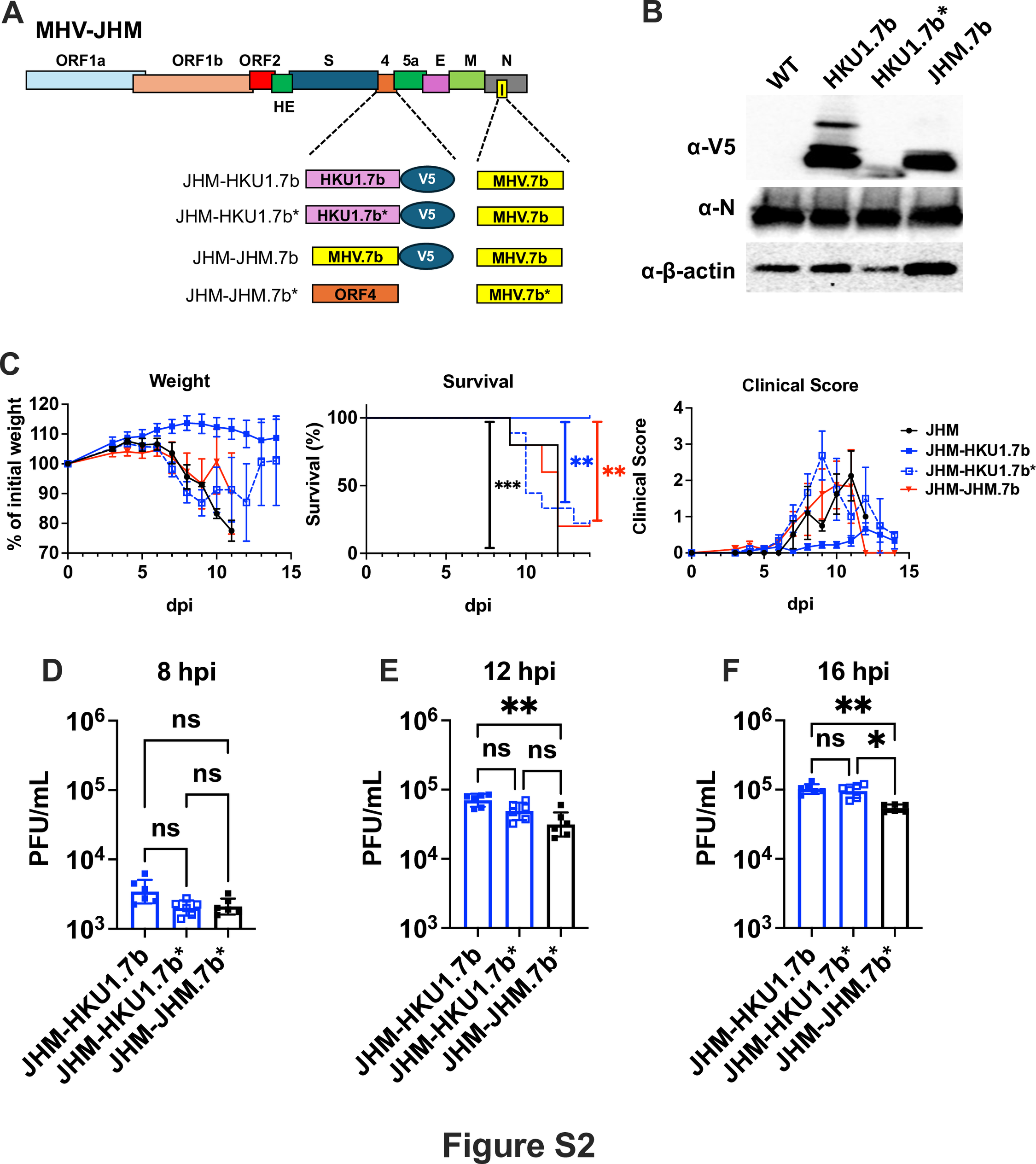

Supplement: S2 Fig — (A) Schematic diagram illustrating the introduction of I protein and the corresponding control sequences to replace ORF 4 of JHM. (B) HeLa-MVR cells were infected with the indicated viruses at a multiplicity of infection (MOI) of 0.01. Infected cells were harvested at 16 hpi. Cell lysates were subject to SDS-PAGE. The I proteins of HCoV-HKU1 and JHM were detected with an anti-V5 antibody (α-V5). Viral nucleocapsid protein (α-N) and β-actin (α-β-actin) were probed to control for virus replication and protein amount, respectively. (C) Percent of initial weight (left panel), survival (middle panel) and clinical scores (right panel) of C57BL/6 mice intranasally infected with 500 PFU of WT JHM (black line), JHM-HKU1.7b (blue solid line), JHM-HKU1.7b* (blue dashed line) or JHM-JHM.7b* (red line). Data are pooled from at least two independent experiments (n ≥ 5 for each group). Data points are shown as mean ± SEM for the weight curve and the panel for clinical score. The P value in the survival curve was determined with logrank (Mantel-Cox) test followed by Bonferroni’s correction for multiple comparisons. The survival curve of JHM-HKU1.7b is significantly different from JHM, JHM-HKU1.7b* and JHM-JHM.7b. Font color represents statistical significance of the correspondingly colored group compared to JHM-HKU1.7b. **P < 0.01, ***P < 0.001. (D-F) BMDM isolated from C57BL/6 mice were infected with JHM-HKU1.7b, JHM-HKU1.7b* or JHM-JHM.7b* at an MOI of 0.01. Infected BMDM were collected for determination of virus titers at 8 (D), 12 (E) and 16 (F) hpi. Data are pooled from two independent experiments. Each point represents data obtained from an individual biological replicate. Data points are shown as geometric mean ± geometric SD. Statistical significance determined by one-way ANOVA with correction for multiple comparisons. *P < 0.05, **P < 0.01. (TIF) [file ppat.1013148.s002.tif]

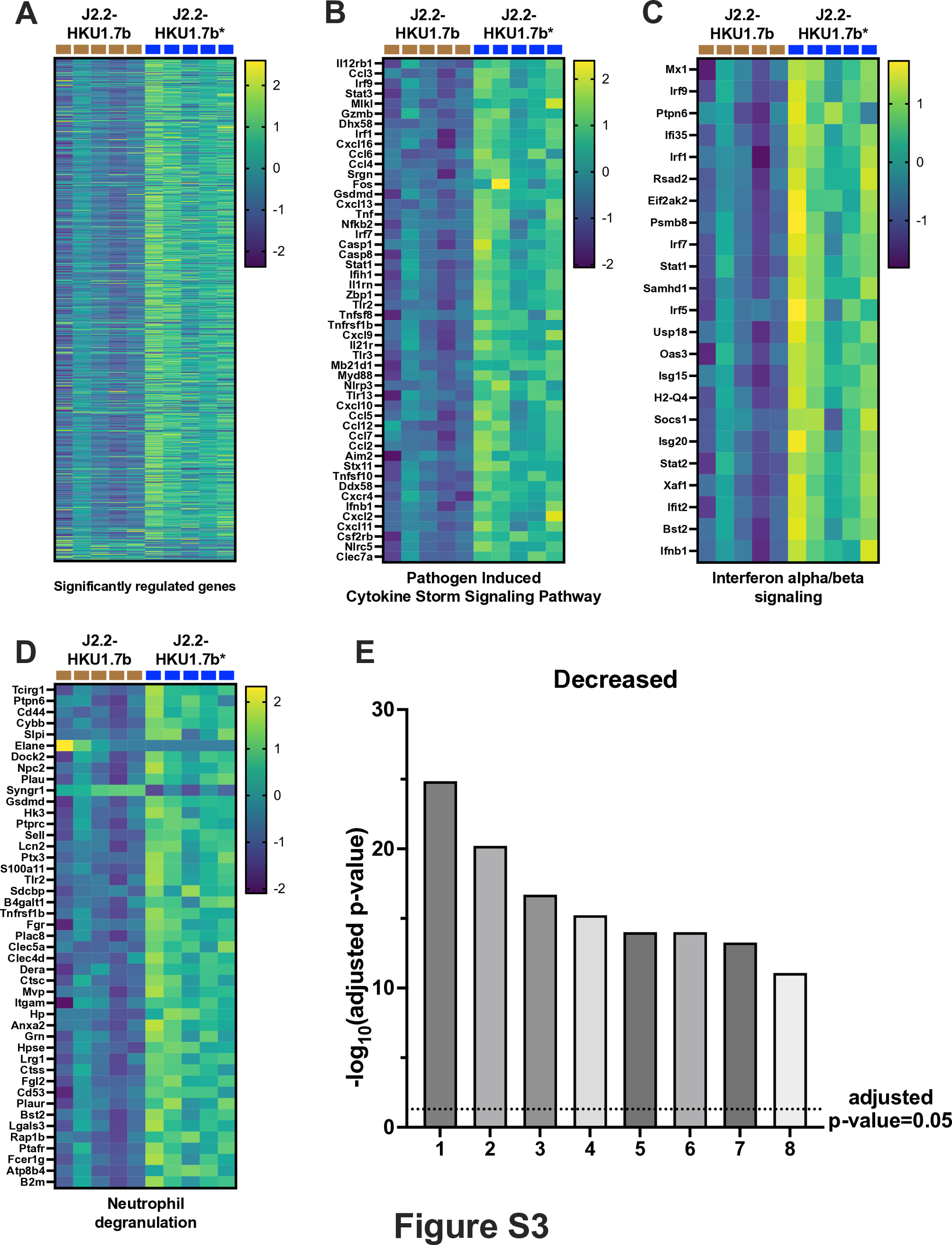

Supplement: S3 Fig — C57BL/6 mice were intracranially infected with 750 PFU of J2.2-HKU1.7b or J2.2-HKU1.7b*. The brains were harvested at 3 dpi for isolation of total RNA followed by RNA sequencing. Genes with adjusted P < 0.05 were selected for further analysis. Heat maps plotting log10 z-score of all significantly regulated genes (A), genes involved in pathogen induced cytokine signaling pathway (B), interferon alpha/beta signaling (C) and neutrophil degranulation (D) at 3 dpi. (E) Ingenuity Pathway Analysis (Qiagen) was used to analyze altered biological pathways (J2.2-HKU1.7b vs. J2.2-HKU1.7b*) at 3 dpi. Pathways with adjusted P < 0.05 are considered significant. 1: Pathogen Induced Cytokine Storm Signaling Pathway; 2: Interferon alpha/beta signaling; 3: Neutrophil degranulation; 4: ISGylation Signaling Pathway; 5: Interferon gamma signaling; 6: Role of PKR in Interferon Induction and Antiviral Response; 7: IL-27 Signaling Pathway; 8: Macrophage Classical Activation Signaling Pathway. (TIF) [file ppat.1013148.s003.tif]

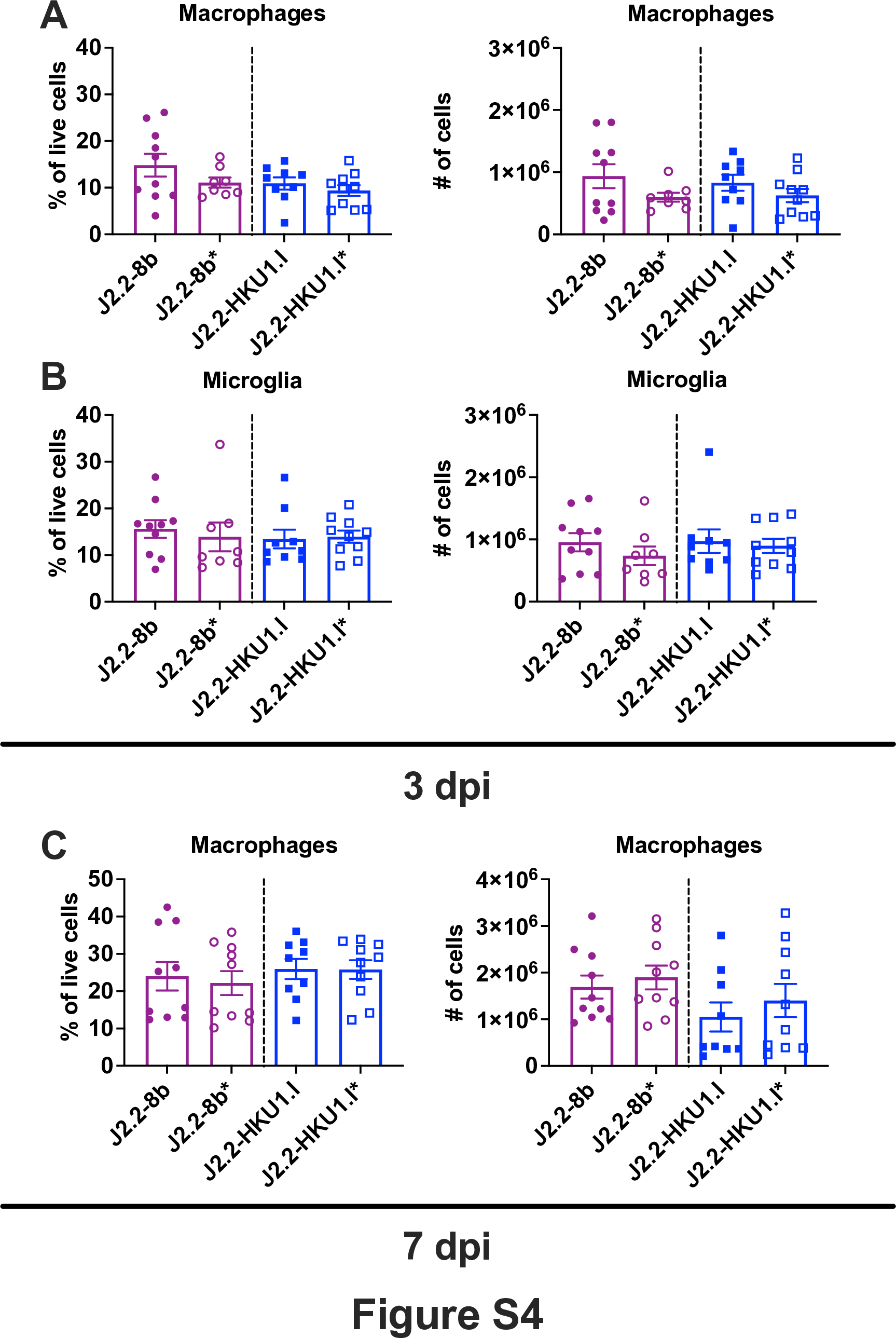

Supplement: S4 Fig — C57BL/6 mice were intracranially infected with 750 PFU of J2.2- MERS.8b, J2.2-MERS.8b*, J2.2-HKU1.7b or J2.2-HKU1.7b*. The brains were harvested at 3 and 7 dpi for flow cytometric analysis of immune cell infiltration. Frequency (left panel) and number (right panel) of macrophages (A), microglia (B) at 3 dpi and macrophages at 7 dpi in infected brains are illustrated. Data are pooled from two independent experiments. Each point represents data obtained from an individual mouse. Data points are shown as mean ± geometric SEM. (TIF) [file ppat.1013148.s004.tif]

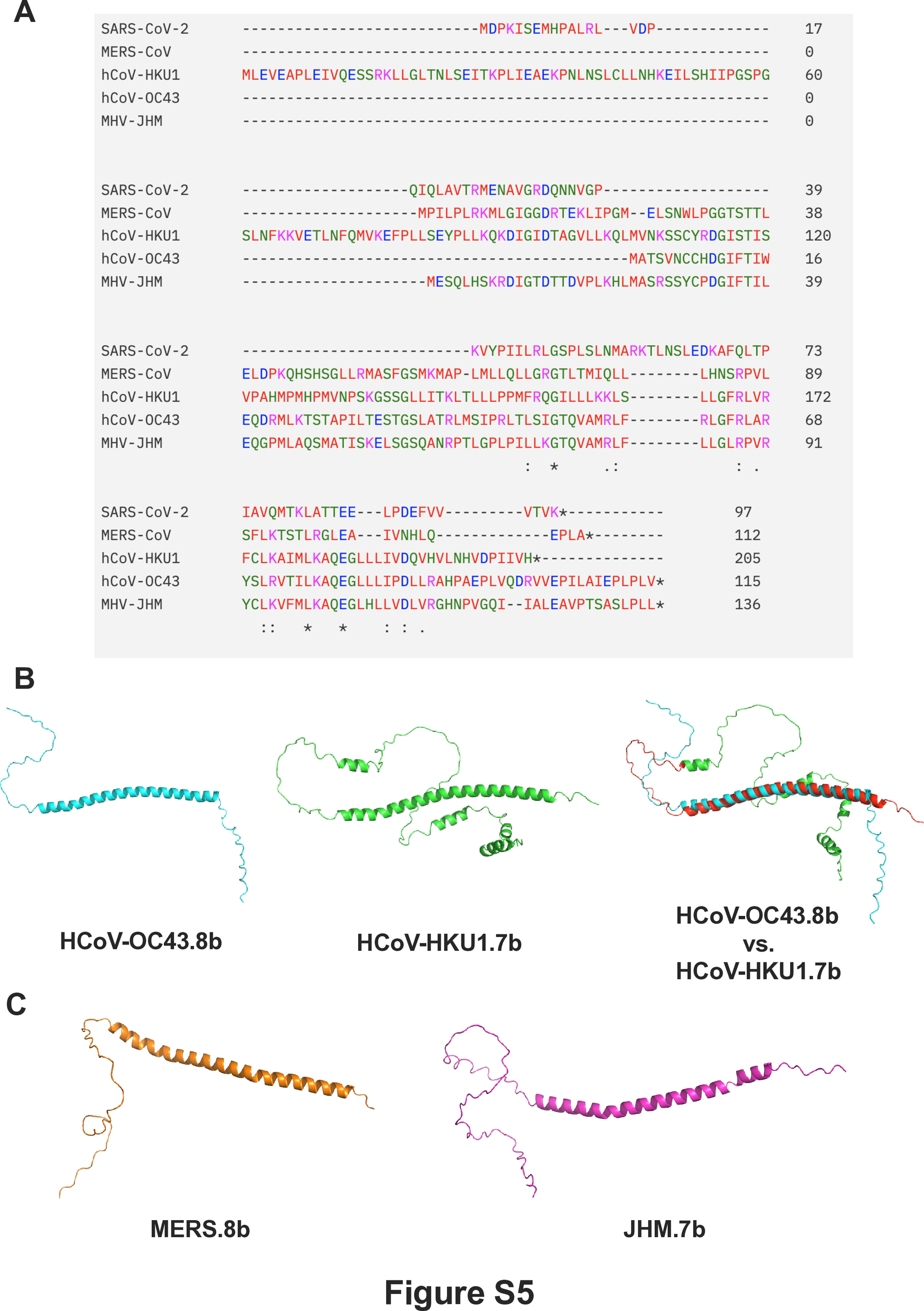

Supplement: S5 Fig — (A) Sequence alignments of the indicated betacoronavirus I protein. (B) Predicted structures of the HCoV-OC43 protein 8b (left) and the HCoV-HKU1 protein 7b (middle) using AlphaFold. Structural homology shown in the superimposed structure (right). (C) Predicted structures of MERS-CoV protein 8b [19] (left) and MHV-JHM [31] (right) obtained from previous studies. (TIF) [file ppat.1013148.s005.tif]

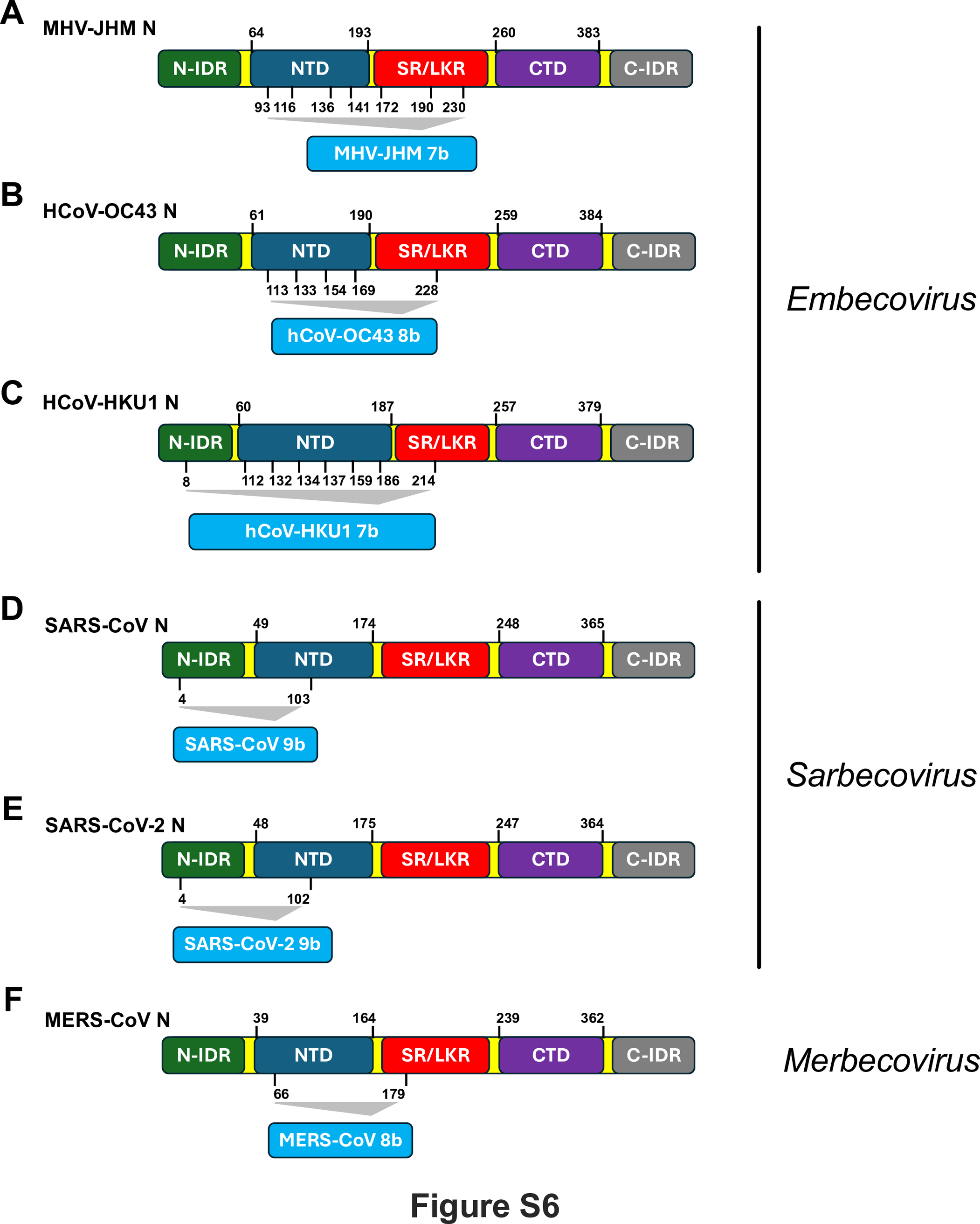

Supplement: S6 Fig — (A-F) The genomic location of the internal genes of representative betacoronaviruses are illustrated relative to the N gene and the corresponding protein domains. The numbers represent the first of the two consecutive codons of the N gene that constitute the corresponding codon of the internal genes (+1 reading frame relative to N). The internal genes are encompassed within the first and last indicated codons of the N gene. The corresponding N protein domains expressed by the N sequences that overlap with internal genes are indicated. The positions of alternative start codons for each internal protein are shown in A-C. N-IDR: N-terminal intrinsically disordered region; NTD: N-terminal domain; SR: Serine/Arginine rich motif; LKR: Linker region; CTD: C-terminal domain; C-IDR: C-terminal intrinsically disordered region. (TIF) [file ppat.1013148.s006.tif]
